# Positional Differences of the Mandibular Canal in Relation to Permanent Mandibular First Molars with Eruption Disturbances in Children

**DOI:** 10.3390/children7110206

**Published:** 2020-10-31

**Authors:** Eungyung Lee, Taesung Jeong, Jonghyun Shin

**Affiliations:** 1 Department of Pediatric Dentistry, Dental Research Institute, Pusan National University Dental Hospital, Yangsan 50612, Korea; leeungyung@gmail.com (E.L.); tsjeong@pusan.ac.kr (T.J.); 2 Department of Pediatric Dentistry, School of Dentistry, Dental and Life Science Institute, Pusan National University, Yangsan 50612, Korea

**Keywords:** eruption disturbance, permanent mandibular first molar, mandibular canal, children, cone-beam computed tomography

## Abstract

Eruption disturbances in permanent mandibular first molars (PM1s) are uncommon. This retrospective study aimed to investigate differences in the position of the mandibular canal in relation to PM1s, with or without, eruption disturbances. Panoramic and cross-sectional views were reconstructed from cone-beam computed tomography imaging of children with PM1 eruption disturbances. Distances from the most inferior margin of the mandible to the center of the mandibular canal (M–C) and from the outer margin of the lingual cortex to the center of the mandibular canal (L–C) were measured for normally erupted PM1s (normal group) and for PM1s with eruption disturbances (ED group) and compared using independent *t*-tests. The mean M–C was significantly shorter in the ED group (4.86 ± 1.07 mm) than in the normal group (6.56 ± 1.06 mm) (*p* < 0.05). The mean L–C was also significantly shorter in the ED group (2.74 ± 0.74 mm) than in the normal group (3.09 ± 0.71 mm) (*p* < 0.05). This study demonstrated that the mandibular canal tended to be positioned more inferiorly in relation to PM1s with eruption disturbances than normally erupted PM1s in children. Clinicians should be aware of this positional deviation when managing children with PM1 eruption disturbances.

## 1. Introduction

Impaction or primary retention of a tooth indicates eruption cessation before emergence, regardless of the presence or absence of a physical barrier in the eruption path [1]. The frequency of eruption disturbances, especially impactions, is the highest for mandibular and maxillary third molars, followed by maxillary canines [2]. Although an impaction can occur in any tooth in the dental arches, impaction of a mandibular first molar is relatively rare, with a reported prevalence of 0.01% to 5% for the permanent mandibular first molars (PM1s) [3,4,5,6]. Eruption disturbances of PM1s can lead to a variety of clinical problems, including root resorption of adjacent teeth, cyst formation, malocclusion due to elongation of the opposite teeth, and decreased vertical dimensions [1]. Therefore, it is of vital importance to detect these eruption disturbances early enough to guide teeth to their appropriate positions, particularly in growing children and adolescents.

Treatment options for eruption disturbances vary depending on etiology, patient age, anticipated treatment compliance, and relative position of the nonerupted tooth within the dental arch. Treatment options include periodic check-ups, surgical exposure, orthodontic traction combined with surgical exposure, surgical repositioning, and extraction [1,7]. Teeth with eruption disturbances and incomplete root development are highly likely to spontaneously erupt as roots develop [8]. If this spontaneous eruption fails to occur, more active interventions, such as surgical exposure or orthodontic traction are inevitable. Surgical repositioning and extractions are generally considered last-line treatment options and are undertaken only if orthodontic traction has failed or if a tooth is positioned too deeply to erupt in a relatively short period of time [1,7]. Prior to undertaking a surgical procedure, however, it is necessary to evaluate the position of key anatomical structures around the targeted tooth. The mandibular canal and inferior alveolar nerve (IAN) are two of the most important anatomical structures in the mandibular molar area, and surgical procedures in this region can potentially lead to IAN injury, resulting in temporary or even permanent sensory impairment [9,10,11].

There have been several previous studies evaluating the positioning of the mandibular canal in relation to the permanent mandibular third molars to reduce the risk of complications [12,13,14,15,16,17]. Other studies have performed radiographic evaluations of the mandibular canal at the level of the mandibular first molars; however, these studies were performed exclusively in patients with mature and normally erupted mandibular first molars [18,19,20,21]. More recently, it has been reported that mandibular cysts can affect the length and position of the mandibular canal [22]. To our knowledge, however, there have been no previous studies examining the position of the mandibular canal in relation to PM1s with eruption disturbances in children. Therefore, this study aimed to compare the position of the mandibular canal in relation to PM1s with or without eruption disturbances using cone-beam computed tomography (CBCT) in a pediatric population.

## 2. Materials and Methods

### 2.1. Ethics Statement

Owing to the retrospective, noninterventional design of this study, a written exemption was granted by the Institutional Review Board (IRB) of Pusan National University Dental Hospital (PNUDH-2019-044). The IRB of the Pusan National University Dental Hospital also waived the requirement for individual informed consent, and thus, written/verbal informed consent was not obtained from participants or their legal guardians. All study data were analyzed anonymously.

### 2.2. Subject Selection

Electronic medical records of patients who visited Pusan National University Dental Hospital between March 2004 and July 2020 with a chief complaint of an eruption disturbance of PM1 were retrospectively reviewed. Medical records of patients whose oral-maxillo-facial readings of panoramic radiographs noted a PM1 impaction during this same timeframe were also reviewed. This cross-sectional study was designed in accordance with the Reporting of Observational Studies in Epidemiology (STROBE) statement [23]. Patients were considered eligible for study inclusion if they were diagnosed with an impaction or primary retention of a PM1 and if they were in the mixed dentition phase with the presence of at least the primary mandibular right and left second molars. Subjects were selected from six to 12 years of age, so as to be expected to cooperate to take appropriate radiographs and obviously diagnosed as an eruption disturbance of a PM1 with at least the primary mandibular second molars.

Patients were excluded from this study if their eruption disturbances were associated with developmental disorders or systemic diseases, maxillofacial deformities, secondary retentions (i.e., a cessation of eruption after emergence), mesial inclinations after passage through the alveolar bone, or locking due to restoration of adjacent primary molars. Patients with permanent dentition, radiographic distortions, or incomplete medical or radiographic records were also excluded. Eruption disturbances in this study were defined as an impaction or primary retention, indicating cessation of tooth eruption prior to emergence of a tooth in the jaw.

### 2.3. CBCT Analysis

CBCT scans were acquired with Pax-Zenith 3D (Vatech Co., Hwaseong, Korea) with the following scanning parameters: 105 kVp, 4 mA, 24 s, voxel size of 0.2 mm, and field of view of 20 × 19 cm. CBCT imaging analysis was performed using OnDemand 3D software (Cybermed Inc., Daejeon, Korea). 

**Figure 1:**
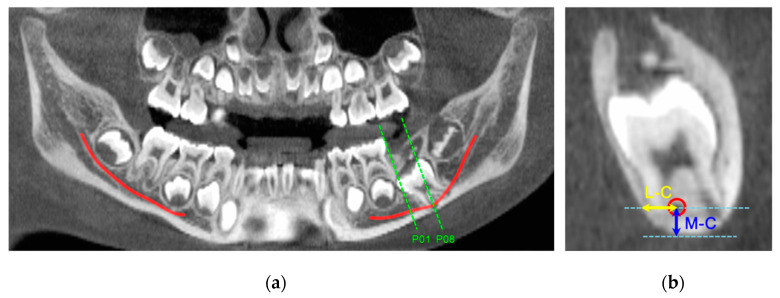
Cone-beam computed tomography (CBCT) imaging of study patients analyzed using OnDemand 3D software. (**a**) Reconstructed panoramic view with nerve tracing in permanent mandibular first molars (PM1) areas. Red lines indicate positions of the mandibular canals on this panoramic view. The green line (noted P01) indicates the first measurement point on the cross-sectional view of this PM1 in the ED group; (**b**) Cross-sectional CBCT image displaying measurement point P08. M–C refers to the distance from the most inferior margin of the mandible to the center of the mandibular canal. L–C refers to the distance from the outer margin of the lingual cortex to the center of the mandibular canal. A red circle indicates the mandibular canal.

A panoramic view was reconstructed by setting the arch based on the contact point of the normally erupted PM1 with the adjacent primary mandibular second molar. The mandibular canal was visualized using the software’s nerve-tracing tool (Figure 1a). A reference line was drawn parallel to the tooth axis of the primary mandibular second molar to obtain a cross-sectional view. Fifteen cross-sectional views with 1 mm intervals were obtained bilaterally from the distal contact point of the primary mandibular second molar to the PM1.

Two anatomical measurements, including the distance from the most inferior margin of the mandible to the center of the mandibular canal (M–C) and the distance from the outer margin of the lingual cortex to the center of the mandibular canal (L–C), were taken (in mm) on each cross-sectional view (Figure 1b). Measurements were performed for normally erupted PM1s (normal group) and for PM1s with eruption disturbances (eruption disturbance group, ED group). Differences in mean values of all 15 measurement points were compared between the two groups. In addition, means of each measurement point were used to trace the mandibular canal in mesiodistal and buccolingual directions. 

### 2.4. Statistical Analysis

All statistical analyses were performed using SPSS ver. 22.0 software (IBM SPSS, Armonk, NY, USA). The chi-squared test was used to identify associations between gender and region of eruption disturbances. Independent *t*-tests were used to determine associations between age and gender and for comparisons of mean values between normal and ED groups. The significance level was set at a *p*-value of < 0.05.

## 3. Results

A total of 42 children’s (24 boys and 18 girls) CBCT data were analyzed in this study. The mean age was 8.2 ± 1.1 years, with no significant differences in age, gender, or left- vs. right-sided PM1 eruption disturbances (Table 1 and Table 2). All PM1 eruption disturbances were unilateral.

**Figure 2:**
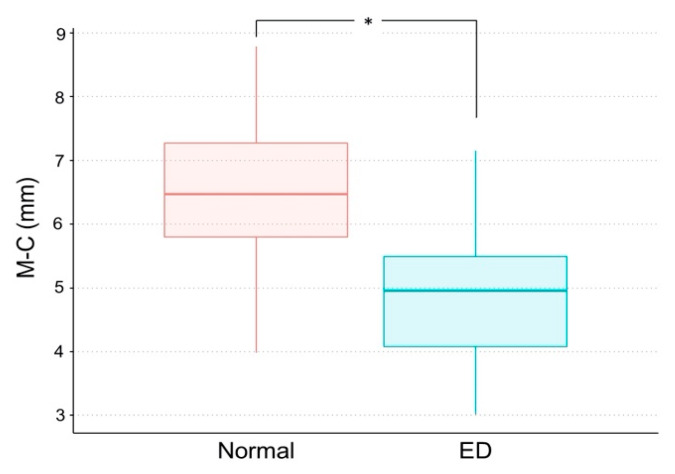
Box plot for comparison of the distance from the most inferior margin of the mandible to the center of the mandibular canal (M–C) in the normal and eruption disturbance (ED) groups. Asterisk (*) indicates a significant difference.

**Figure 3:**
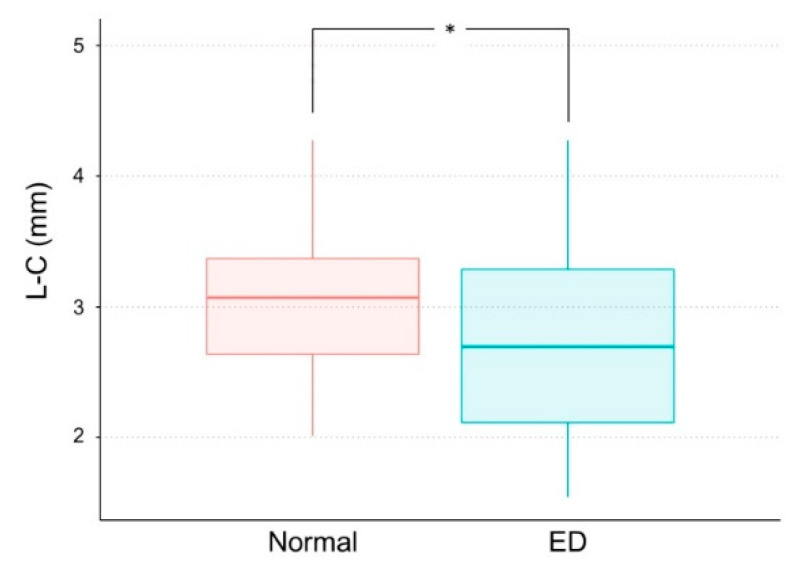
Box plot for comparison of the distance from the outer margin of the lingual cortex to the center of the mandibular canal (L–C) in the normal and eruption disturbance (ED) groups. Asterisk (*) indicates a significant difference.

**Figure 4:**
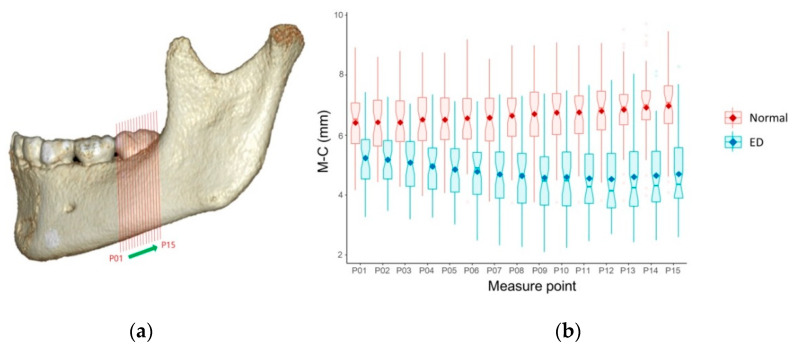
Mesiodistal position of the mandibular canal in relation to permanent mandibular first molar (PM1). (**a**) Reconstructed lateral view of the mandible with PM1 and measurement points; (**b**) Box plots for comparison of mean values of the distance from the most inferior margin of the mandible to the center of the mandibular canal (M–C) at each measurement point in the normal and eruption disturbance (ED) groups. The dots in the center of the boxes are mean values. These values indicate the mesiodistal position of the mandibular canal at each measurement point.

**Figure 5:**
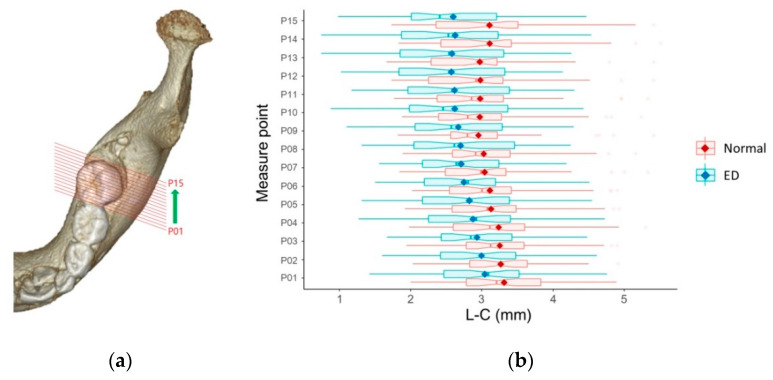
Buccolingual position of the mandibular canal in relation to PM1. (**a**) Reconstructed axial view of mandible with PM1 and measurement points; (**b**) Box plots for comparison of mean values of the distance from the outer margin of the lingual cortex to the center of the mandibular canal (L–C) at each measurement point in the normal and eruption disturbance (ED) groups. The dots in the center of the boxes are mean values. These values indicate the buccolingual position of the mandibular canal at each measurement point.

The mean M–C was significantly shorter in the ED group (4.86 ± 1.07 mm) than in the normal group (6.56 ± 1.06 mm) (*p* < 0.05) (Table 3, Figure 2). The mean L–C was also significantly shorter in the ED group (2.74 ± 0.74 mm) than in the normal group (3.09 ± 0.71 mm) (*p* < 0.05) (Table 3, Figure 3). Figure 4 and Figure 5 show box plots for comparisons of mean M–C and L–C values obtained at each measurement point in the normal and ED groups.

## 4. Discussion

Early identification and timely interventions for eruption disturbances are of paramount importance in school-aged children to avoid subsequent issues related to occlusion and maxillofacial growth. It is also vitally important to understand the anatomy of areas surrounding eruption disturbances, as injuries to these regions during treatment can lead to serious complications. In this study, we performed quantitative analyses of CBCT imaging data to compare the position of the mandibular canal in relation to PM1s with or without eruption disturbances in children. We observed that the mandibular canal was positioned more lingually and inferiorly in relation to PM1s without eruption than PM1s with normal eruption. 

**Figure 6:**
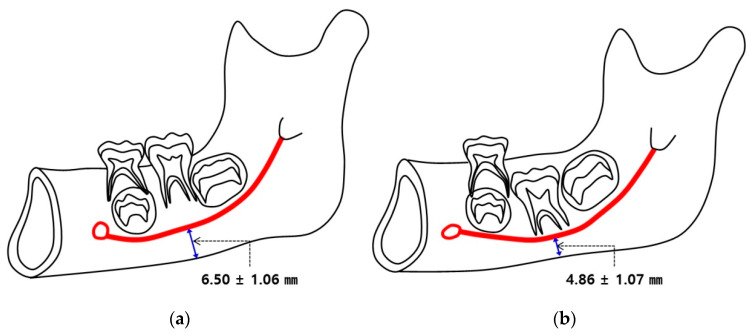
Schematic drawings displaying results of this study. The mandibular canal tends to be positioned more inferiorly in relation to permanent mandibular first molars with eruption disturbances than with normal eruption. (**a**) mean value of the distance from the most inferior margin of mandible to center of mandibular canal (M–C) in normal group; (**b**) mean value of the distance from the most inferior margin of mandible to center of mandibular canal (M–C) in eruption disturbance (ED) group.

In adults, the mandibular canal is generally located almost 10 mm above the inferior border of the mandible [24]. In this study, we demonstrated that the mean distance from the most inferior margin of the mandible to the center of the mandibular canal in the normal group was 6.50 ± 1.06 mm, a value similar to what has been reported in the Indian population [25]. In contrast, this mean distance was 4.86 ± 1.07 mm in the ED group. Figure 6 shows schematic drawings of the mean distances between the mandibular canal and the inferior border of the mandible in the normal versus ED groups, demonstrating displacement of the mandibular canal in children with PM1 eruption disturbances. 

In a study of Korean children and adolescents, the typical sequence of tooth eruption was reported for mandibular first molars and anterior teeth. In that study, the mean age of mandibular permanent tooth eruption was reported to be 6.22 years and 6.12 years for boys and girls, respectively [26]. In the present study, the mean age of included subjects was 8.2 ± 1.1 years, likely because most study patients were referred from private dental clinics after periodic check-ups, regardless of whether molars had erupted spontaneously. Additionally, eruption disturbances in some patients were only detected through radiographic examinations, with some patients not even aware of non-erupted teeth. 

Eruption disturbances can either be caused by local factors, such as odontomas, supernumerary teeth, or cysts, or by systemic diseases like cleidocranial dysplasia, Crouzon syndrome, or rickets [1,7]. While systemic factors generally affect multiple teeth, local factors usually affect only one or a few teeth [1,7]. As all PM1 eruption disturbances were unilateral in our study patients, it can be assumed that they were primarily caused by local factors, such as odontomas or dentigerous cysts. We also explicitly excluded patients with systemic diseases, syndromes, or maxillofacial deformities in our study design. Indeed, we identified odontomas above six impacted PM1s out of the 42 PM1s with eruption disturbances included in this study. In nine patients, developmental disorders of teeth, including delayed development or congenital absence of mandibular second molars, peg lateralis, or impactions of maxillary canines, were also observed. We could not, however, verify significant associations between PM1 impactions and developmental or eruptive disorders of other teeth owing to the small number of subjects included in the study. 

The mandibular canal is an anatomical structure within the mandible containing the IAN, artery, and vein [20,27]. The IAN can be observed in the mandibular process by approximately 5 weeks after conception [28]. The relative position of the mandibular canal in the mandible changes during a child’s functional progression from suckling to the early stages of mastication [29]. As masticatory muscle activity stimulates bone growth, growth of the buccal cortical bone in the mandible moves the mandibular canal relatively more lingually [29]. Further, the position of the mandibular canal within the mandible varies depending on age, gender and race [21]; however, the relative position of the mandibular canal at the levels of the mandibular foramen, PM1, and mental foramen tend to remain constant regardless of age or gender [30]. Yeh et al. also reported that there were no significant differences in the distance from the IAN to the IBM (inferior border of the mandible) on the left or right sides, indicating that the location of the mandibular canal tends to be similar bilaterally if the mandibular shape is symmetrical and if there are no eruptive disorders of mandibular molars [31]. 

Prior to performing a surgical intervention in the mandibular molar region, it is important to identify the location of the mandibular canal to reduce the risk of IAN injuries. The incidence of IAN injuries associated with impacted mandibular third molars is 0.4–13.4% [32,33]. Without a thorough knowledge of the surrounding anatomy, the risk of postoperative complications, including sensory impairments like paresthesias or dysesthesias, may increase. Therefore, preoperative imaging evaluations to determine the position of the mandibular canal should precede surgical interventions for PM1 eruption disturbances in children. Some roots of the PM1s with eruption disturbances were dilacerated enclosing the mandibular canal in the present study. The more the roots of the PM1s with eruption disturbances are severely dilacerated and enclose the mandibular canal, the more confinement of the mandibular canal will be received. There is a lack of knowledge about the vitality of a PM1 with the eruption disturbance. It can be assumed that the restricted mandibular canal may cause sensory and motor problems associated with complications of IAN injury.

Unfortunately, there have been very few previous studies focused on eruption disturbances of PM1s in children [34,35], with most reports focused on describing successful strategies for eruption in PM1s with disturbances [36,37,38,39]. Etiologies for eruption disorders have also not yet been clearly elucidated. The causal relationship between the eruption disturbance of a PM1 and the deviation of mandibular canal could not be clarified in this study. On the basis of a similar study, it may be reasonable to consider the positional change of the mandibular canal as a result [22]. If the deviated mandibular canal was the result from the eruption disturbance of a PM1, it implies that the eruption disturbances of PM1s can affect to the growth and development of the mandible and also the rear part of the PM1s. The delayed development or the congenital missing of the permanent mandibular second molar can advocate this perspective as previously mentioned.

Owing to limitations of this cross-sectional study, we could not identify when mandibular canal deviations began in these children. Moreover, it was also not possible to determine the extent to which jaw growth and dentition transitions influenced positional changes of the mandibular canal in these children. It has not yet been verified whether a deviated mandibular canal can recover its position after growth and development in children. Therefore, future studies related to the timing and etiology of mandibular canal displacements at eruption disturbance sites are necessary.

## 5. Conclusions

This study analyzed positional differences of the mandibular canal in relation to PM1s with or without eruption disturbances, demonstrating that the mandibular canal tended to be positioned more inferiorly in relation to PM1s with eruption disturbances than PM1s with normal eruption. Therefore, clinicians should be aware of this potential positional deviation when managing children with PM1 eruption disturbances, particularly when planning surgical interventions. With this knowledge, the incidence of complications related to IAN injury may be reduced. 

## Figures and Tables

**Table 1:** Distribution of gender and age of children in this study.

Gender	*n*	Mean Age ± SD	*p*-Value
Boys	24	8.5 ± 0.9	0.065
Girls	18	7.8 ± 1.2	
Total	42	8.2 ± 1.1	

**Table 2:** Distribution of impacted molars with respect to gender.

	Boys (*n*)	Girls (*n*)	Total	*p*-Value
#36 ^1^	11	5	16	0.084
#46 ^2^	13	13	26	
Total	24	18	42	

^1^ #36: permanent mandibular left first molar with eruption disturbances. ^2^ #46: permanent mandibular right first molar with eruption disturbance.

**Table 3:** Minimum, maximum, and mean measurements of distances from the center of the mandibular canal to the lingual and inferior cortices of the mandible.

Variable		*n*	Mean ± SD (mm)	Minimum (mm)	Maximum (mm)	*p*-Value
M–C ^1^	ED ^3^	42	4.86 ± 1.07	3.01	7.15	0.000 *
	Normal	42	6.50 ± 1.06	3.98	8.78	
L–C ^2^	ED ^3^	42	2.74 ± 0.74	1.54	4.27	0.027 *
	Normal	42	3.09 ± 0.71	2.01	5.03	

^1^ M–C: distance from the most inferior margin of the mandible to the center of the mandibular canal. ^2^ L–C: distance from the outer margin of the lingual cortex to the center of the mandibular canal. ^3^ ED: eruption disturbance * Significant using an independent *t*-test at *p* < 0.05.
